# Determinants of biventricular cardiac function: a mathematical model study on geometry and myofiber orientation

**DOI:** 10.1007/s10237-016-0825-y

**Published:** 2016-08-31

**Authors:** Marieke Pluijmert, Tammo Delhaas, Adrián Flores de la Parra, Wilco Kroon, Frits W. Prinzen, Peter H. M. Bovendeerd

**Affiliations:** 1 Department of Biomedical Engineering, Cardiovascular Research Institute Maastricht, Maastricht University, Universiteitssingel 50, P.O. Box 616, 6200 MD Maastricht, The Netherlands; 2 Department of Physiology, Cardiovascular Research Institute Maastricht, Maastricht University, Universiteitssingel 50, P.O. Box 616, 6200 MD Maastricht, The Netherlands; 3 Department of Biomedical Engineering, Eindhoven University of Technology, P.O. Box 513, 5600 MB Eindhoven, The Netherlands; 4 Institute of Computational Science, University of Lugano, Lugano, Switzerland

**Keywords:** Adaptation, Computational physiology, Finite element, Biventricle

## Abstract

In patient-specific mathematical models of cardiac electromechanics, usually a patient-specific geometry and a generic myofiber orientation field are used as input, upon which myocardial tissue properties are tuned to clinical data. It remains unclear to what extent deviations in myofiber orientation and geometry between model and patient influence model predictions on cardiac function. Therefore, we evaluated the sensitivity of cardiac function for geometry and myofiber orientation in a biventricular (BiV) finite element model of cardiac mechanics. Starting out from a reference geometry in which myofiber orientation had no transmural component, two new geometries were defined with either a 27 % decrease in LV short- to long-axis ratio, or a 16 % decrease of RV length, but identical LV and RV cavity and wall volumes. These variations in geometry caused differences in both local myofiber and global pump work below 6 %. Variation of fiber orientation was induced through adaptive myofiber reorientation that caused an average change in fiber orientation of ${\sim }8^\circ$ predominantly through the formation of a component in transmural direction. Reorientation caused a considerable increase in local myofiber work $({\sim }18\,\%)$ and in global pump work $({\sim }17\,\%)$ in all three geometries, while differences between geometries were below 5 %. The findings suggest that implementing a realistic myofiber orientation is at least as important as defining a patient-specific geometry. The model for remodeling of myofiber orientation seems a useful approach to estimate myofiber orientation in the absence of accurate patient-specific information.

## Introduction

In the last decade, several biventricular (BiV) finite element (FE) models have been developed as a step toward model-assisted clinical decision-making in cardiac disease (Aguado-Sierra et al. 2011; Gurev et al. 2011; Niederer et al. 2011; Sermesant et al. 2012). These models take into account the heart’s geometry, myofiber orientation, and myocardial tissue properties. Whereas geometrical characteristics can be captured from imaging data and included into models through sophisticated meshing algorithms (Lamata et al. 2014), determination of local myofiber orientation and material properties is more challenging. Usually generic data from in vitro DTMRI or histological measurements (LeGrice et al. 1997; Nielsen et al. 1991) are morphed into geometrically correct models (Peyrat et al. 2007; Vadakkumpadan et al. 2012). Next, myocardial tissue properties are tuned for maximum agreement between model predicted and clinically measured cardiac function (Aguado-Sierra et al. 2011; Niederer et al. 2011). In principle, the estimates of these tissue properties are affected by the deviations between the generic and the (unknown) patient-specific field of myofiber orientation.

**Fig. 1:**
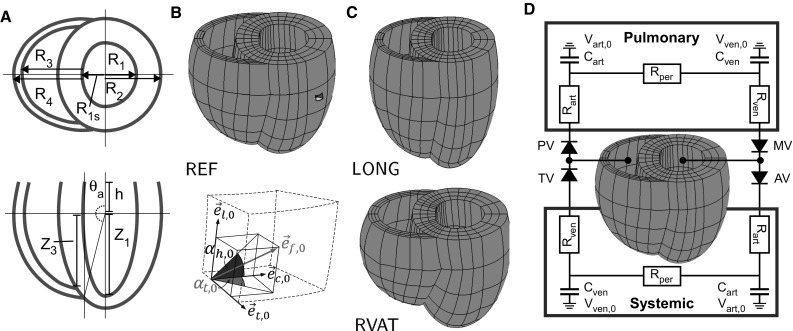
The model of BiV mechanics. **a** Illustration of geometrical parameters, used in Table 1, in cross sections of the REF geometry. **b** FE mesh of simulation REF , and myofiber orientation vector in the unloaded state $\vec {e}_{f,0}$ quantified by helix angle $\alpha _{h,0}$ and transverse angle $\alpha _{t,0}$ using a local cardiac coordinate system $\{\vec {e}_{t,0},\, \vec {e}_{l,0},\, \vec {e}_{c,0}\}$. **c** Elongated mesh (LONG) and a mesh with a higher attachment of the right to the left ventricle (RVAT). **d** Lumped parameter model of the circulation. The valves are modeled as ideal diodes: *AV* aortic valve, *MV* mitral valve, *PV* pulmonary valve, *TV* tricuspid valve. Both pulmonary and systemic circulation are modeled using compliances and resistances: $C_{art}$, arterial compliance; $C_{ven}$, venous compliance; $R_{art}$, arterial resistance; $R_{per}$, peripheral resistance; $R_{ven}$, venous resistance; $V_{art,0}$, zero-pressure arterial volume; $V_{ven,0}$, zero-pressure venous volume. Parameter values are listed in Table 2

The accuracy of DTMRI is on the order of $\pm 10^\circ$ (Reese et al. 1995), even in the in vitro case (Hsu et al. 1998; Scollan et al. 1998). This accuracy seems to be sufficient for modeling cardiac electrophysiology (Vadakkumpadan et al. 2012). However, in models of left ventricular (LV) mechanics it has been shown that variations in the spatial distribution of myofiber orientations of $8^\circ$ introduced variations in myofiber stress of 10 % (Geerts-Ossevoort et al. 2003). In the same model, a drastic geometrical variation in the short- to long-axis ratio from 1:1 to 1:5 yielded similar variations in myofiber stress of 10 %. However, these results might not be valid in a BiV model, among others because of the nonsymmetrical BiV shape and the attachment of the right ventricle (RV) to the LV.

The aim of this study was to investigate the sensitivity of cardiac function to geometry and myofiber orientation in a BiV FE model of cardiac mechanics. First, local myofiber function and global pump function were compared between three geometries, which differed only by either a 27 % difference in LV short- to long-axis ratio or a 16 % difference in RV length. Next, in each of these three models the sensitivity for myofiber orientation was tested by letting the myofiber orientation change according to an adaptive myofiber reorientation model. We evaluated sensitivity to geometry and fiber orientation by assessing global function, local function, and cardiac deformation.

## Methods

### Geometry

In the passive stress-free state, the BiV geometry was described by two intersecting, truncated ellipsoids (Fig. 1), defined by 8 input parameters (Table 1). Left ventricular wall volume $V_{lvw}$ determines the overall size, whereas dimensionless parameters $f_V,f_{sep},f_R,f_h,f_T,f_{VRL}$, and $\theta _A$ determine BiV shape. Parameter values were chosen such that the geometry for the reference simulation REF was representative for an average healthy human heart (Ho and Nihoyannopoulos 2006).

**Table 1:** Biventricular geometry input parameter values

Parameter	Definition	Value	Unit	Explanation
$V_{lvw}$		160	[ml]	LV wall volume
$f_V$	$V_{lv,0}/V_{lvw}$	0.375	[-]	LV cavity-to-wall volume ratio in the stress-free state
$f_{VRL}$	$V_{rv,0}/V_{lv,0}$	1.0	[-]	RV-to-LV cavity volume ratio
$f_R$	$R_1/(h+Z_1)$	0.55 (0.40)$^\mathrm{a}$	[-]	Endocardial LV radius-to-length ratio
$f_h$	$h/Z_1$	0.40	[-]	Truncation height above equator-to-LV endocardial length ratio
$f_{sep}$	$R_{1s}/R_1$	0.70	[-]	Septal endocardial radius $(R_{1s})$-to-LV endocardial radius $(R_1)$ ratio
$f_T$	$\frac{R_2-R_1}{R_4-R_3}$	0.33 (0.35)$^\mathrm{b}$	[-]	LV-to-RV wall thickness ratio
$\theta _A$		$0.85\pi \,(0.75\pi )^\mathrm{b}$	[rad]	Lowest attachment angle of RV to LV

See Fig. 1a for definition of *R*’s, *Z*’s, *h*, and $\theta _A$

$^\mathrm{a}$ The value between brackets holds for simulation LONG

$^\mathrm{b}$ The values between brackets hold for simulation RVAT

### Myofiber orientation

Myofiber orientation in the unloaded state $\vec {e}_{f,0}$ was defined in a local cardiac coordinate system $\{\vec {e}_{t,0},\, \vec {e}_{l,0},\, \vec {e}_{c,0}\}$ by two angles (Fig. 1b). The helix angle $\alpha _{h,0}$ was defined as the angle between $\vec {e}_{c,0}$ and the projection of $\vec {e}_{f,0}$ on the circumferential-longitudinal plane $(\vec {e}_{c,0},\, \vec {e}_{l,0})$. The transverse angle $\alpha _{t,0}$  was defined as the angle between $\vec {e}_{c,0}$ and the projection of $\vec {e}_{f,0}$ on the circumferential-transmural plane $(\vec {e}_{c,0},\vec {e}_{t,0})$. We used the bidirectional spherical linear interpolation method of Bayer et al. (2012) to ensure a smooth transition of the coordinate system and myofiber orientation in all regions, including the RV-LV attachment.

During simulation of the cardiac cycle, deformation of the tissue caused a change from the reference orientation $\vec {e}_{f,0}$ into an actual myofiber orientation $\vec {e}_f$. In addition, $\vec {e}_{f,0}$ was subject to adaptation that caused a structural change in myofiber orientation in the unloaded reference state as proposed by Kroon et al. (2009):

1 $$\begin{aligned} \frac{\partial \vec {e}_{f,0}}{\partial t} = \frac{1}{\kappa }(\vec {e}^*_{f}-\vec {e}_{f,0}) \end{aligned}$$

with an adaptation time constant $\kappa$ that was set to four times the cardiac cycle time. $\vec {e}^*_f$ is the actual myofiber orientation in the deformed tissue corrected for rigid body rotation:

2 $$\begin{aligned} \vec {e}_f = \frac{\varvec{F}\cdot \vec {e}_{f,0}}{\lambda _{f}} = \frac{\varvec{R}\cdot \varvec{U}\cdot \vec {e}_{f,0}}{\lambda _{f}} = \varvec{R}\cdot \vec {e}^*_f \quad \hbox {with} \quad \lambda _{f} = |\varvec{U}\cdot \vec {e}_{f,0}| \end{aligned}$$

with $\lambda _f$ the myofiber stretch ratio and $\varvec{F}$ the deformation gradient tensor that consists of the deformation $\varvec{U}$ and rigid body rotation $\varvec{R}$. In nodes on the endo- and epicardial surfaces, the myofiber vector resulting from adaptation was modified by projecting it onto the surface, to ensure that myofibers do not stick out of these surfaces.

### Material properties

Myocardial tissue Cauchy stress $\varvec{\sigma }$ was composed of a passive component $\varvec{\sigma }_p$ and an active component $\sigma _a$:

3 $$\begin{aligned} \varvec{\sigma } = \varvec{\sigma }_p + \sigma _a\vec {e}_f \vec {e}_f \end{aligned}$$

Constitutive relations for $\varvec{\sigma }_p$ and $\sigma _a$ were identical to those used by Kerckhofs et al. (2003). Passive material behavior was assumed nonlinearly elastic, transversely isotropic, and nearly incompressible, with parameter settings identical to those used by Kerckhofs et al. (2003). Active stress $\sigma _a$ depends on time elapsed since activation $t_a$, sarcomere length $l_s$, and sarcomere shortening velocity, with parameter settings identical to those used by Bovendeerd et al. (2009). Active stress development was initiated simultaneously throughout both ventricles with a cycle time of 800 ms.

### Governing equations and boundary conditions

In the model, the quasi-static equations of conservation of linear momentum were solved:

4 $$\begin{aligned} \vec {\nabla }\cdot \varvec{\sigma }=\vec {0} \end{aligned}$$

with $\vec {\nabla }$ the spatial gradient operator. At the base, rotation of the endocardial basal ring and movement of the whole basal plane in normal direction were prevented. The epicardial surface was assumed to be traction free while the RV and LV endocardial surfaces were uniformly subjected to a right and left ventricular pressure, $p_{rv}$ and $p_{lv}$, respectively. During isovolumic contraction (IC) and relaxation (IR) phases of the cardiac cycle, $p_{rv}$ and $p_{lv}$ were determined such that mechanical equilibrium of the myocardial tissue was obtained at a constant end-diastolic or end-systolic RV and LV volume, respectively. During the filling and ejection phase, $p_{rv}$ and $p_{lv}$ were computed from the interaction of the RV and LV with lumped parameter models of the pulmonary and systemic circulation, respectively (Fig. 1d) (Bovendeerd et al. 2009). Parameter values of the resistances and compliances were chosen such that they are representative for characteristics of the human circulation (Table 2) (Shoukas 1975).

**Table 2:** Parameter values for the pulmonary and systemic circulation

Parameter	Definition	Unit	Systemic	Pulmonary
$R_{art}$	Outflow resistance	$[\hbox {kPa}\,\hbox {s}\,\hbox {ml}^{-1}]$	$4.46\times 10^{-3}$	$2.48\times 10^{-3}$
$R_{per}$	Peripheral resistance	$[\hbox {kPa}\,\hbox {s}\,\hbox {ml}^{-1}]$	$1.49\times 10^{-1}$	$1.78\times 10^{-2}$
$R_{ven}$	Inflow resistance	$[\hbox {kPa}\,\hbox {s}\,\hbox {ml}^{-1}]$	$1.10\times 10^{-3}$	$2.18\times 10^{-3}$
$C_{art}$	Arterial compliance	$[\hbox {ml}\,\hbox {kPa}^{-1}]$	$1.53\times 10^{1}$	$4.59\times 10^{1}$
$C_{ven}$	Venous compliance	$[\hbox {ml}\,\hbox {kPa}^{-1}]$	$4.59\times 10^{1}$	$1.53\times 10^{1}$
$V_{art,0}$	Arterial unstressed volume	[ml]	$7.04\times 10^{2}$	$7.83\times 10^{1}$
$V_{ven,0}$	Venous unstressed volume	[ml]	$3.16\times 10^{3}$	$5.13\times 10^{2}$
$V_{tot}$	Total blood volume	[ml]	$5.00\times 10^{3}$	

### Numerical implementation

The equilibrium equations (4) were solved numerically with a Galerkin-type FE method using 27-noded hexahedral elements with a tri-quadratic interpolation of the displacement field. The mesh consisted of 684 elements and 19803 degrees of freedom. The time step was fixed at 1 ms. It took about 2 h to compute one cardiac cycle on a single core of an Intel Core i7-2600 processor on a personal computer.

### Simulations performed

The sensitivity of cardiac function to geometry was investigated using three different geometries with identical LV wall volume of 160 ml and RV wall volume of 40 ml. In the unloaded state, both LV and RV cavity volume were 60 ml. With respect to the reference geometry REF (Fig. 1b; Table 1), parameter $f_R$ was decreased from 0.55 to 0.40 to create a more elongated geometry LONG (Fig. 1c, top). Geometry RVAT was obtained by changing $\theta _A$ from $0.85\pi$ to $0.75\pi$, thus shifting the attachment of the RV toward the base (Fig. 1c, bottom). To maintain the same RV wall volume, the LV-to-RV wall thickness ratio $f_T$ was changed from 0.33 to 0.35.

In all geometries, the initial distribution of $\alpha _{h,0}$ varied nonlinearly with the transmural position from $65^\circ$ at the endocardium to $-50^\circ$ at the epicardium (Bovendeerd et al. 2009). The initial condition for $\alpha _{t,0}$  was set to zero. Still, a transmural component in myofiber orientation is already observed near the attachment regions (see dashed lines in septal long-axis cross sections in Fig. 4) as a result of the interpolation method (Bayer et al. 2012).

The sensitivity of cardiac function to myofiber orientation was investigated by changing myofiber orientation through adaptive reorientation. The first 12 consecutive cardiac cycles were used to approximate a hemodynamic steady state while myofiber reorientation was disabled. In the subsequent 20 cardiac cycles, myofiber orientation was adapted per node according to the adaptation model (Eq. 1). Adaptation was disabled in the final 5 cardiac cycles, during which a steady hemodynamic state was reached again.

Results from the three geometries were analyzed in terms of global pump function and local myofiber function in both the initial state (averaged over cycles 8–12) and the adapted state (averaged over cycles 33–37). Global function was quantified as cardiac output (CO), LV ejection fraction (EF), and stroke work *W*. Local function was quantified as natural myofiber strain during isovolumic contraction $\epsilon _{f,ic}$, during ejection $\epsilon _{f,ej}$, and during isovolumic relaxation $\epsilon _{f,ir}$, as well as by maximum active myofiber Cauchy stress $\sigma _{f,max}$, and stroke work density $w_f$ calculated from the area within the active myofiber Cauchy stress–natural strain loop. Local function variables are presented as mean and standard deviation (SD) calculated from the nodes in the four LV and septal cross sections, indicated in the mesh in Fig. 4. We also evaluated myofiber angles $\alpha _{h,0}$ and $\alpha _{t,0}$  in the adapted state. Finally, we compared model computed with experimentally determined circumferential-radial shear $E_{cr}$, referred to the state at begin ejection (Bovendeerd et al. 2009; Delhaas et al. 2008; Pluijmert et al. 2014; Ubbink et al. 2006).

**Fig. 2:**
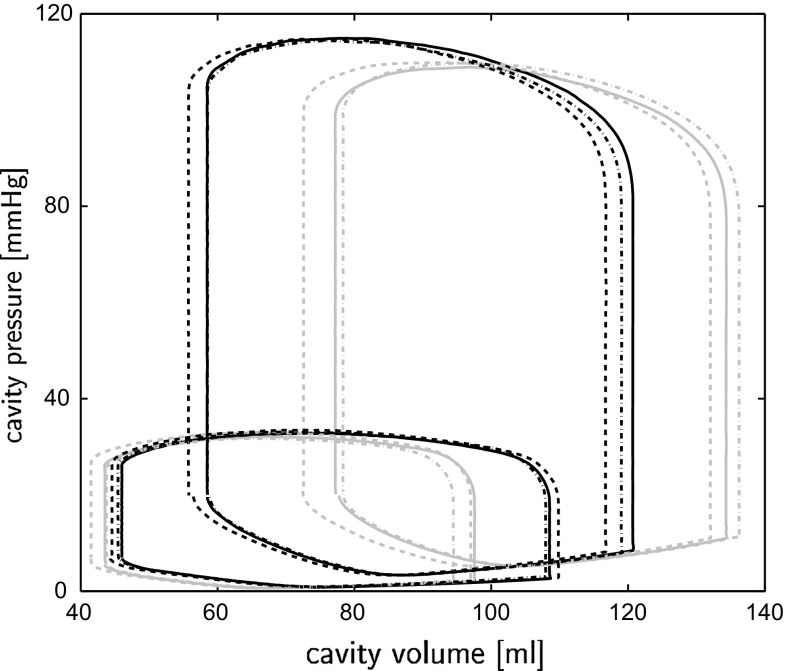
Pressure–volume loops in the three geometries REF (*solid*), LONG (*dashed*), and RVAT (*dashed-dotted*), in the initial state (*gray*) and the adapted state (*black*)

**Table 3:** Global function presented as LV stroke work $W_{lv}$, RV stroke work $W_{rv}$, cardiac output CO, and LV and RV ejection fraction EF

	REF		LONG		RVAT	
	Init	Adap	Init	Adap	Init	Adap
$W_{lv}$[J]	0.73	0.85	0.77	0.84	0.74	0.83
$W_{rv}$[J]	0.21	0.24	0.22	0.25	0.19	0.24
CO [l/min]	4.19	4.70	4.34	4.74	4.09	4.63
LV EF [-]	0.43	0.52	0.45	0.52	0.43	0.51
RV EF [-]	0.55	0.58	0.57	0.60	0.54	0.58

**Table 4:** Local function presented as $(\hbox {mean}\pm \hbox {SD})$ natural myofiber during isovolumic contraction $\epsilon _{f,ic}$, during ejection $\epsilon _{f,ej}$, and during isovolumic relaxation $\epsilon _{f,ir}$, as well as maximum active myofiber Cauchy stress $\sigma _{f,max}$, and stroke work density $w_f$. Mean and SD were calculated from the septal and LV free wall nodes indicated in Fig. 4

		REF		LONG		RVAT	
		Init	Adap	Init	Adap	Init	Adap
$\epsilon _\mathrm{f,ic}$	[%]	−3.9 (2.0)	−1.1 (1.6)*	−3.2 (1.8)	−1.4 (1.3)*	−3.8 (2.0)	−1.3 (1.5)*
$\epsilon _\mathrm{f,ej}$	[%]	−12.5 (1.6)	−14.3 (1.6)*	−12.8 (1.4)	−13.8 (1.5)*	−12.6 (1.5)	−13.6 (1.4)*
$\epsilon _\mathrm{f,ir}$	[%]	3.2 (2.0)	1.2 (1.6)*	3.1 (1.8)	1.9 (1.3)*	3.3 (2.0)	1.5 (1.5)*
$\sigma _\mathrm{f,max}$	[kPa]	43 (13)	46 (12)	44 (12)	46 (10)	43 (13)	46 (12)
$w_\mathrm{f}$	[kPa]	4.8 (1.8)	5.8 (1.7)*	5.2 (1.7)	5.9 (1.6)*	5.0 (1.8)	5.7 (1.7)*

* Indicates statistically significant change between initial and adapted state

## Results

In the initial state, only small differences in global pump function were present between geometries (Fig. 2; Table 3). For the three geometries REF, LONG  and RVAT  mean (range) values were 0.75 (0.73–0.77) J for LV stroke work $W_{lv}$, 0.21 (0.19–0.22) J for RV stroke work $W_{rv}$, 4.21 (4.09–4.34) l/min for cardiac output CO, 0.44 (0.43–0.45) for LV ejection fraction EF, and 0.56 (0.54–0.57) for RV EF. Differences in mean myofiber function between geometries were small as well (Table 4).

Adaptive myofiber reorientation caused a change in myofiber orientation of about $8^\circ$ in all three geometries. It also caused an increase of global function (Fig. 2; Table 3): In simulation, REF, LONG  and RVAT  $W_{lv}$  increased by 16, 9, and 11 %, $W_{rv}$  by 16, 17, and 25 %, CO by 12, 9, and 13 %, LV EF by 21, 15, and 19 %, and RV EF by 4, 4 and 8 %, respectively. Mean myofiber function improved as well (Table 4): Myofiber shortening during ejection increased by 15, 9, and 11 %, $\sigma _{f,max}$  increased by 4, 4, and 5 %, and $w_f$ increased by 18, 14, and 18 %, respectively. Absolute myofiber strain during the isovolumic phases decreased. All improvements in local function were statistically significant $(p<0.01)$, except for the increase of $\sigma _{f,max}$  in simulation REF(*p* = 0.057), LONG (*p* = 0.021), and RVAT (*p* = 0.013). Differences in local function were not statistically different in between geometries with adapted myofiber orientation.

**Fig. 3:**
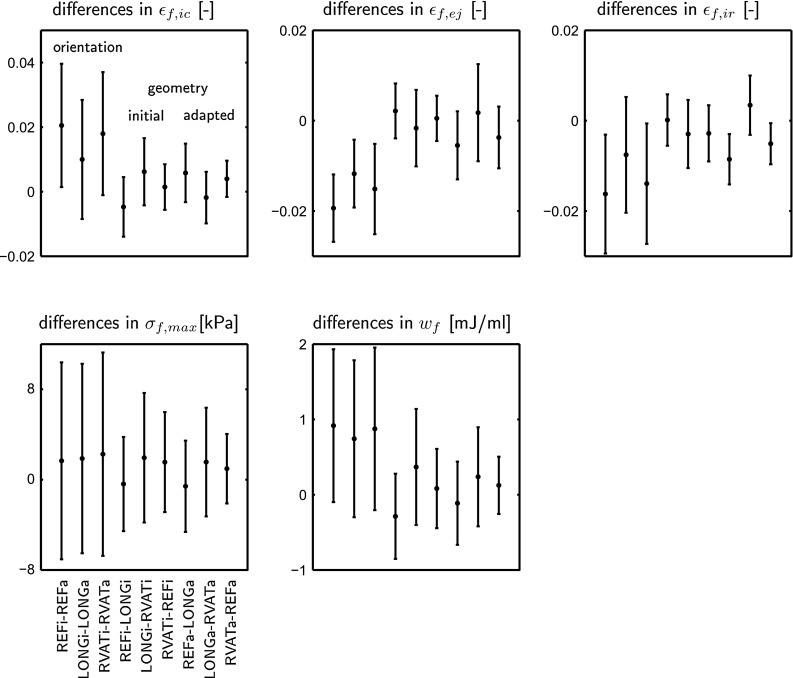
Mean $(\pm \hbox {SD})$ differences between corresponding nodes in the mesh (see Fig. 4) of local function variables: myofiber strain during isovolumic contraction ($\epsilon _{f,ic}$), during ejection ($\epsilon _{f,ej}$), and during isovolumic relaxation ($\epsilon _{f,ir}$), maximum myofiber stress ($\sigma _{f,max}$), and stroke work density $(w_f)$. Results represent differences between the initial and adapted state in the three geometries (REFi-REFa, LONGi-LONGa, RVATi-RVATa), differences between geometries in the initial state (REFi-LONGi, LONGi-RVATi, RVATi-REFi), and differences between geometries in the adapted state (REFa-LONGa, LONGa-RVATa, RVATa-REFa)

Figure 3 shows the mean ± SD of the differences in local function variables between corresponding nodal points in the initial and adapted state, and between corresponding nodal points in different geometries. Increase of myofiber strain during ejection (top row, middle panel) due to reorientation is significantly different from zero for all three geometries (first three values). To a lesser extent, this also holds for changes in myofiber strain during the isovolumic phases, and for $w_f$, but not for $\sigma _{f,max}$. In both the initial and adapted state, differences between geometries are not significantly different from zero (last six values).

**Fig. 4:**
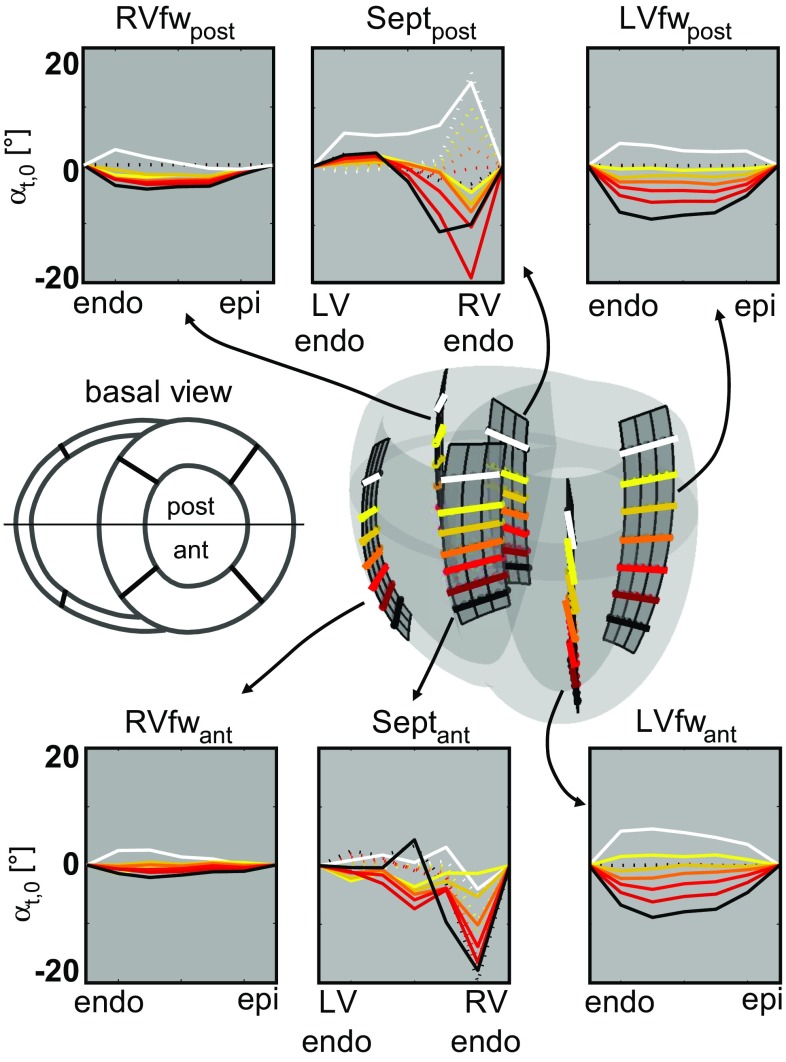
Distributions of the transverse angle $\alpha _{t,0}\,[^\circ ]$ in the initial (*dashed*) and adapted (*solid*) state in the REF simulation, displayed for two cross sections in the LV free wall at the anterior (LVfw$_{ant}$) and posterior (LVfw$_{post}$) side, two in the septum (Sept$_{ant}$ and Sept$_{post}$), and two in the RV free wall (RVfw$_{ant}$ and RVfw$_{post}$). In each cross section, results at 7 levels between apex and base are shown

In the three geometries, the change in myofiber orientation was similar not only in magnitude, but also in pattern. The helix angle $\alpha _{h,0}$ hardly changed, implying that the change in orientation took place especially in the transmural direction. Results on the transverse angle $\alpha _{t,0}$  are shown in detail in Fig. 4 for simulation REF. In the LV free wall, a gradient in $\alpha _{t,0}$  developed with negative values near the apex and positive values near the base. Largest values for $\alpha _{t,0}$  developed in the septum, especially at the RV endocardial side. In the basal region of the septum, $\alpha _{t,0}$  was positive at the posterior side, but about zero at the anterior side. To a lesser extent, asymmetry in myofiber orientation between anterior and posterior side was also seen in the LV and RV free wall. In the RV free wall, $\alpha _{t,0}$  showed similar patterns as in the LV free wall, but angles are smaller compared to the LV free wall and septum.

**Fig. 5:**
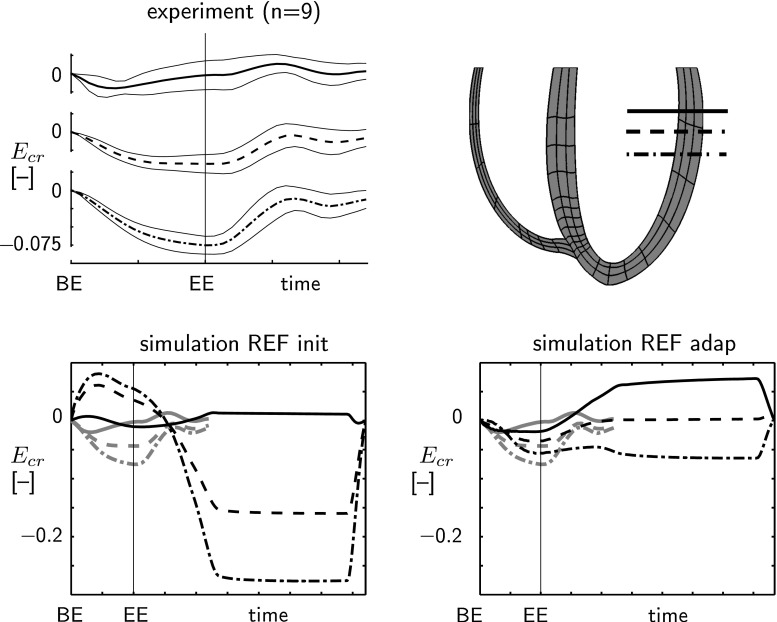
Comparison of experimental and model computed time course of circumferential-radial shear $E_{cr}$. Average $E_{cr}$ $(\hbox {mean}\pm \hbox {SD})$ from nine healthy subjects as measured with MRT (Delhaas et al. 2008) is shown on the *top left*; BE = begin ejection; EE = end ejection; the location of the MR-slices relative to the model geometry at begin ejection is shown on the *top right*. The *bottom row* shows model computed $E_{cr}$ in the REF simulation in the initial state (*left*) and in the adapted state (*right*); model computed and experimental $E_{cr}$ are indicated by the *black* and the *gray* tracings, respectively

Temporal variations in circumferential-radial shear $E_{cr}$ in simulation REF are shown in Fig. 5. In the experimental results (Pluijmert et al. 2014), the total amplitude of $E_{cr}$ is ${\sim }0.10$ and the gradient in $E_{cr}$ at end ejection is apex–mid–base. In the initial state in the model, the total amplitude of $E_{cr}$ is $\sim$0.35 with a base–mid–apex gradient at end ejection. In the adapted state, the total amplitude reduced to $\sim$0.15 and the gradient at end ejection flipped to apex–mid–base, which agrees better with the experimental data. Still, clear differences remain between patterns of $E_{cr}$ in model and experiment. Again, a similar trend was observed in simulations LONG  and RVAT.

## Discussion

We investigated the sensitivity of cardiac function for geometry and myofiber orientation in a BiV FE model of cardiac mechanics. Geometrical variations in the LV short- to long-axis ratio of 27 % (in simulation LONG) or in the RV length by 16 % (in simulation RVAT) resulted in a change of total pump work of only 5 or 0 %, respectively. On the other hand, a change in myofiber orientation over an average angle of about $8^\circ$, as induced by adaptive myofiber reorientation (Eq. 1), caused an increase in total pump work of about 17 % in REF, 11 % in LONG  and 14 % in RVAT.

### Model setup

We used a parameterized description of the BiV geometry, since it enabled well-defined variations of the geometry while keeping wall and cavity volumes constant. This was important, since it is known that the ratio of cavity-to-wall volume largely determines the mean level of active wall stress (Arts et al. 1991). We also started out from a parameterized description of the myofiber orientation. While it seems tempting to vary orientation by adapting coefficients in this description, we considered such variations rather arbitrary. By using the evolution equation (1), we applied a less arbitrary, and presumably more physiologically based variation. Finally, values of the venous compliances, $C_{s,ven}$ and $C_{p,ven}$ were reduced tenfold in comparison with reported experimental data (Shoukas 1975). This choice lowered the typical time constant of the circulation model and thus decreased the number of cycles needed to reach a hemodynamic steady state. Since the same circulation model was used in all simulations, we expect that this modification did not affect the results of our sensitivity study.

### Relation between myofiber orientation and cardiac function

To explain the increase in LV hemodynamic function, we consider sarcomere length in the LV and Sept nodes, indicated in Fig. 4. In model REF at end filling, sarcomere length is smaller in the adapted state $(2.16\pm 0.05\,\upmu {\text{ m }})$ than in the initial state $(2.18\pm 0.05\,\upmu \mathrm{m})$, in agreement with the lower end-diastolic volume in the adapted state (Fig. 2). During the isovolumic contraction (IC) phase ($\epsilon _{f,ic}$), myofiber shortening is much lower in the adapted state $(0.02\pm 0.03\,\upmu \mathrm{m})$ than in the initial state $(0.08\pm 0.04\,\upmu {\text{ m }})$. Thus, sarcomere length at begin ejection is higher in the adapted state $(2.14\pm 0.07\,\upmu {\text{ m }})$ than in the initial state $(2.10\pm 0.09\,\upmu {\text{ m }})$, enabling a stronger contraction because of the Frank–Starling mechanism. This causes an increased ejection pressure in the adapted state, causing an increase in arterial blood volume. Since total blood volume is constant, this leads to a decrease in venous blood volume, which causes the reduction of end-diastolic filling pressure and volume, observed in Fig. 2. The reduced myofiber shortening during IC, as caused by the change in myofiber orientation, is reflected also in a lower $E_{cr}$ during IC. The latter agrees better with experimental data: Although these data do not contain the IC phase, the results in Fig. 5 suggest that little $E_{cr}$ occurs during IC, as the signals tend to go back to zero toward the end of filling.

### Remodeling of myofiber reorientation

The initial distribution for $\alpha _{h,0}$  was based on a distribution that was already optimized for homogeneity of myofiber strain during ejection in the LV (Rijcken et al. 1999). This explains why changes in $\alpha _{h,0}$  between the initial and adapted state were small. To investigate the properties of the adapted state, six additional simulations were performed, in which the adapted fiber orientation in model REF was globally changed: We increased and decreased $\alpha _{h,0}$ by $5^\circ$, we multiplied $\alpha _{h,0}$ by 1.1 and 0.9, and we multiplied $\alpha _{t,0}$  by 1.1 and 0.9. These variations all reduced global and local function (on average 2 % reduction in both pump work and myofiber shortening during ejection) and increased standard deviations of local function parameters, reflecting a decrease in homogeneity. This indicates that the solution from the adaptation model is well defined.

Experimental verification of the adapted distribution of $\alpha _{h,0}$ and $\alpha _{t,0}$  is difficult because of the lack and inaccuracy of experimental data. A difference in myofiber orientation of $8^\circ$ can hardly be detected with in vitro, let alone with *in vivo* DTMRI (Scollan et al. 1998; Tseng et al. 1999). The adapted distribution of the helix angle $\alpha _{h,0}$ falls within the admittedly wide range of experimental data. Model predictions of the developed $\alpha _{t,0}$  in the LV free wall and differences in $\alpha _{t,0}$  in between the anterior and posterior septum (Fig. 4) are supported by results from DTMRI studies (Geerts-Ossevoort et al. 2002; Helm et al. 2005). Since we found circumferential-radial shear strain $E_{cr}$ to be very sensitive to myofiber orientation, we used $E_{cr}$ as an indirect measure to test fiber orientation. The better match between model computed and experimentally determined $E_{cr}$ in the adapted state, and the finding that the adapted fiber field is well-defined, suggest that myofiber reorientation could be considered as a method to implement myofiber orientation in (geometrically correct) BiV FE models.

### Toward patient-specific modeling

In recent BiV patient-specific FE models, generic data on myofiber orientation were used. By tuning model parameters other than myofiber orientations, good agreement was reported between model computed and experimental data on hemodynamic function (Sermesant et al. 2012). Still, as not only shown in our study but also suggested from other studies (Carapella et al. 2014), a match in hemodynamic function is no guarantee for correctly predicting deformation. Deviations in circumferential-radial and circumferential-longitudinal shear strain from experimental observations (Carapella et al. 2014) might result from the absence of a transverse component in myofiber orientation. The model of reorientation of myofiber orientation might be used as a method to implement myofiber orientation in (geometrically correct) BiV FE models.

### Study limitations

The results from our model study are dependent on material properties, in particular properties affecting shear deformation. We used a transversely isotropic constitutive model for the passive material that cannot capture the orthotropic behavior that arises from the structural organization of the myocardial tissue in sheets (LeGrice et al. 1997). It has also been shown that including active stress development perpendicular to the myofiber direction influences the amplitudes of the developed $\alpha _{t,0}$, but not the gradient of $\alpha _{t,0}$  after reorientation (Pluijmert et al. 2014). The effect of sheet orientation and orthotropy needs further investigation. Results of the adapted state were analyzed after 20 cardiac cycles with adaptive myofiber reorientation. At this stage, myofiber reorientation did not yet reach a steady state: Myofiber orientation still changed by about $1^\circ$ per cardiac cycle. Because local and global function did not change anymore, we considered the situation after 20 cycles of adaptation representative for the effect of adaptation. Finally, experimental verification of our results on sensitivity of cardiac function to geometry and fiber orientation is difficult, since this would require the ability to select real hearts that differ only in either geometry or fiber orientation. Still, since we only studied a limited number of variations of geometry and fiber orientation, an extended sensitivity analysis might be performed to check our results.

## Conclusion

This model study shows that a geometrical variation of 27 % in the LV short- to long-axis ratio or a variation of 16 % in RV length leads to a change in total pump work of about 5 %, whereas an average change in myofiber orientation of about $8^\circ$ causes an increase in total pump work of 11–19 % in all three geometries. Differences in total pump work between the three geometries with adapted myofiber orientation are below 5 %. These findings indicate the importance of a realistic choice of myofiber orientation. As current techniques for measurement of myofiber orientation lack sufficient accuracy, the model for remodeling of myofiber orientation seems a useful approach to set myofiber orientations in BiV FE models.
